# Supplementary material for: Mutations in PCYT2 disrupt etherlipid biosynthesis and cause a complex hereditary spastic paraplegia
Source: Brain. 2019 Oct 22;142(11):3382–97. doi: 10.1093/brain/awz291 (PMC6821184; doi:10.1093/brain/awz291)
Supplement: awz291_Supplementary_Data [file awz291_supplementary_data.zip › awz291-Suppl_data/Supplementary_Data4.zip!/Supplementary information 3 - fibroblast lipidomics.zip!/Fibroblast lipidomics/Controle vs Patient1/Statistical plots and data/HexCer[d] species/Volcano plot_alternative.pdf]

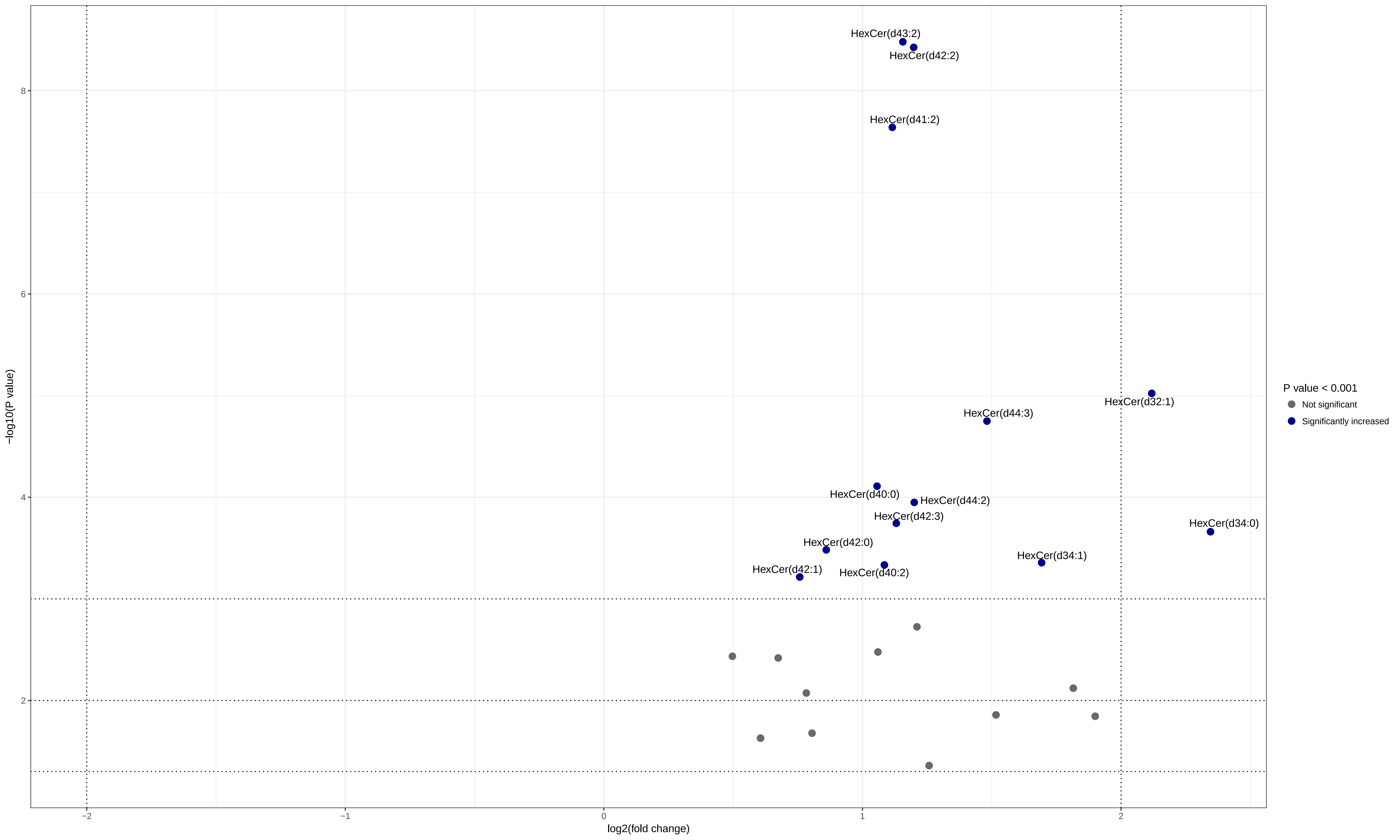
HexCer(d43:2)
HexCer(d42:2)
HexCer(d41:2)
HexCer(d32:1)
HexCer(d44:3)
HexCer(d40:0)
HexCer(d44:2)
HexCer(d42:3)
HexCer(d34:0)
HexCer(d42:0)
HexCer(d34:1)
HexCer(d42:1)
HexCer(d40:2)
P value < 0.001
Not significant
Significantly increased
−log10(P value)
log2(fold change)
8
6
4
2
−2
−1
0
1
2
